# Exercise Training and Neurodegeneration in Mitochondrial Disorders: Insights From the Harlequin Mouse

**DOI:** 10.3389/fphys.2020.594223

**Published:** 2020-12-08

**Authors:** Miguel Fernández-de la Torre, Carmen Fiuza-Luces, Pedro L. Valenzuela, Sara Laine-Menéndez, Joaquín Arenas, Miguel A. Martín, Doug M. Turnbull, Alejandro Lucia, María Morán

**Affiliations:** ^1^Mitochondrial and Neuromuscular Diseases Laboratory, Instituto de Investigación Sanitaria Hospital ‘12 de Octubre’ (‘imas12’), Madrid, Spain; ^2^Physiology Unit, Department of Systems Biology, University of Alcalá, Madrid, Spain; ^3^Spanish Network for Biomedical Research in Rare Diseases (CIBERER), U723, Madrid, Spain; ^4^Wellcome Centre for Mitochondrial Research, Institute of Neuroscience, The Medical School Newcastle University, Newcastle upon Tyne, United Kingdom; ^5^Faculty of Sport Sciences, European University of Madrid, Madrid, Spain; ^6^Spanish Network for Biomedical Research in Fragility and Healthy Aging (CIBERFES), Madrid, Spain

**Keywords:** *Harlequin* mouse, mitochondrial diseases, neurodegeneration, OXPHOS disorders, training

## Abstract

**Aim:**

Cerebellar neurodegeneration is a main phenotypic manifestation of mitochondrial disorders caused by apoptosis-inducing factor (AIF) deficiency. We assessed the effects of an exercise training intervention at the cerebellum and brain level in a mouse model (Harlequin, *Hq*) of AIF deficiency.

**Methods:**

Male wild-type (WT) and *Hq* mice were assigned to an exercise (Ex) or control (sedentary [Sed]) group (*n* = 10–12/group). The intervention (aerobic and resistance exercises) was initiated upon the first symptoms of ataxia in *Hq* mice (∼3 months on average) and lasted 8 weeks. Histological and biochemical analyses of the cerebellum were performed at the end of the training program to assess indicators of mitochondrial deficiency, neuronal death, oxidative stress and neuroinflammation. In brain homogenates analysis of enzyme activities and levels of the oxidative phosphorylation system, oxidative stress and neuroinflammation were performed.

**Results:**

The mean age of the mice at the end of the intervention period did not differ between groups: 5.2 ± 0.2 (WT-Sed), 5.2 ± 0.1 (WT-Ex), 5.3 ± 0.1 (*Hq*-Sed), and 5.3 ± 0.1 months (*Hq*-Ex) (*p* = 0.489). A significant group effect was found for most variables indicating cerebellar dysfunction in *Hq* mice compared with WT mice irrespective of training status. However, exercise intervention did not counteract the negative effects of the disease at the cerebellum level (*i.e.*, no differences for *Hq*-Ex vs. *Hq*-Sed). On the contrary, in brain, the activity of complex V was higher in both *Hq* mice groups in comparison with WT animals (*p* < 0.001), and *post hoc* analysis also revealed differences between sedentary and trained *Hq* mice.

**Conclusion:**

A combined training program initiated when neurological symptoms and neuron death are already apparent is unlikely to promote neuroprotection in the cerebellum of *Hq* model of mitochondrial disorders, but it induces higher complex V activity in the brain.

## Introduction

Mitochondrial disorders (MD) are caused by inherited alterations in genes involved in the oxidative phosphorylation (OXPHOS) system (Ng and Turnbull, 2016). Despite their low prevalence in the general population, these alterations represent the most frequent genetic metabolopathies (Tucker et al., 2011). Numerous pathogenic mutations have been found to cause MD and are frequently associated with severe phenotypic manifestations, notably at the neuromuscular level (Bird et al., 2014), such as those affecting the *AIFM1* gene encoding apoptosis-inducing factor (AIF). The clinical manifestations of patients with AIF deficiency are broad and frequently include ataxia, but the molecular underpinnings for these remain unclear (Bano and Prehn, 2018). In this context, the Harlequin (*Hq*) mouse – a preclinical model of MD harboring a proviral insertion in *Aifm1* causing an ∼80% decrease in AIF expression – has provided some mechanistic insight into the biological alterations leading to cerebellar degeneration (Klein et al., 2002; Vahsen et al., 2004; Bénit et al., 2008).

Although no effective cure is yet available for MD, there is a rationale to support that regular physical exercise can be an adjuvant treatment to attenuate some phenotypic manifestations associated with these conditions, notably the decline in cardiorespiratory and muscle fitness that is commonly found in affected individuals (Cejudo et al., 2005; Taivassalo et al., 1996, 1998, 1999, 2001, 2006; Adhihetty et al., 2007; Murphy et al., 2008; Jeppesen et al., 2006, 2009; Siciliano et al., 2000, 2012; Bates et al., 2013; Fiuza-Luces et al., 2018a). Beyond its well-documented fitness benefits for virtually all population groups, regular exercise might also have potential neuroprotective effects including improving central nervous system (CNS) blood flow and cognitive function, and preventing neurodegeneration and cognitive decline (Delezie and Handschin, 2018; Liu-Ambrose et al., 2018; Cabral et al., 2019; Valenzuela et al., 2020). While more research is needed, there is preliminary evidence supporting that exercise training might also attenuate, at least partly, the cerebellar degeneration associated with some diseases, and thus potentially alleviate ataxic symptoms (Aranca et al., 2016; Ayvat et al., 2018; Oliveira et al., 2018). In this regard, although there is evidence that exercise training – including aerobic exercises alone or in combination with resistance (strength) training – can improve cardiorespiratory fitness and/or muscle mass/strength in patients with MD (Cejudo et al., 2005; Taivassalo et al., 1996, 1998, 1999, 2001, 2006; Adhihetty et al., 2007; Murphy et al., 2008; Jeppesen et al., 2006, 2009; Siciliano et al., 2000, 2012; Bates et al., 2013; Fiuza-Luces et al., 2018a) and in preclinical models of MD (Clark-Matott et al., 2015; Safdar et al., 2011, 2016; Fiuza-Luces et al., 2019; Ross et al., 2019), the evidence regarding potential neuroprotective effects is more scarce, with data supporting aerobic training-induced neuroprotection reported only in the mitochondrial DNA *Mutator* mouse (Safdar et al., 2011; Clark-Matott et al., 2015; Ross et al., 2019). Further, compared to aerobic training the neuroprotective effects of resistance exercise have been scarcely studied (Pinho et al., 2019). This is an important point because it is widely accepted by international guidelines that muscle resistance training should be included in any exercise program for its multi-systemic benefits (World Health Organization, 2010), which are comparable to those of aerobic exercise at least at the cardiometabolic level (Fiuza-Luces et al., 2018b).

The aim of the present study was to assess the effects of an 8-week exercise training intervention (‘Ex,’ including both endurance and resistance exercises) in *Hq* and age- and gender-matched wild-type (WT) mice compared to a control (non-exercise) sedentary (‘Sed’) group, analyzing the currently known hallmarks of disease in the *Hq* mice at the cerebellum level: OXPHOS deficiency, neuronal loss, oxidative stress and neuroinflammation; and at the brain level: analysis of enzyme activities and levels of the oxidative phosphorylation system, oxidative stress and neuroinflammation.

## Results

The mean age of the mice (all four groups combined) at the start of the intervention was 12 ± 0.1 weeks. The mean age of the *Hq* mice (both *Hq*-Ex and *Hq*-Sed group combined) at symptom onset (and thus at the start of the exercise intervention for *Hq*-Ex) was also 12.1 ± 0.1 weeks. The mean age of the mice at the end of the intervention period did not differ between the experimental groups: 5.2 ± 0.2 (WT-Sed), 5.2 ± 0.1 (WT-Ex), 5.3 ± 0.1 (*Hq*-Sed), and 5.3 ± 0.1 months (*Hq*-Ex) (*p* = 0.489).

### OXPHOS System

**FIGURE 1:**
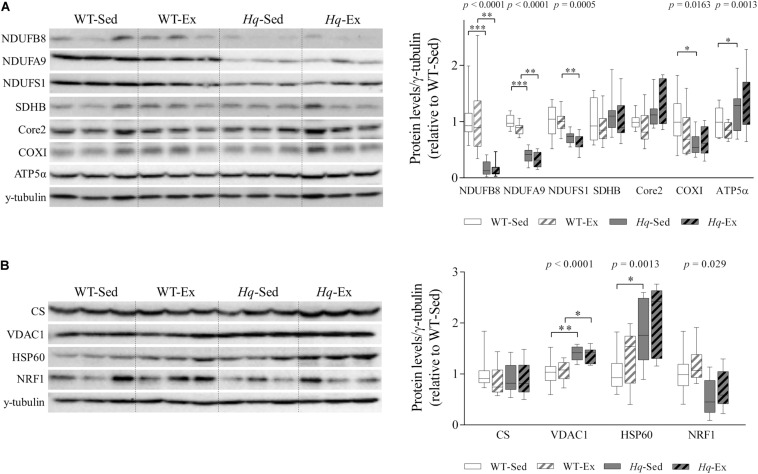
OXPHOS subunits and mitochondrial content. **(A)** Western blot analysis of representative OXPHOS components and **(B)** mitochondrial content markers in cerebellum homogenates of wild-type sedentary (WT-Sed, *n* = 12) and exercise-trained (WT-Ex, *n* = 11) mice, and of *Hq* sedentary (*Hq*-Sed, *n* = 11) and exercise-trained (*Hq*-Ex, *n* = 10) mice. Densitometry analysis: protein levels were normalized to γ-tubulin levels; data (median, interquartile range, and min and max values) are expressed relative to levels in the WT-Sed group. Significant *p-*values for group effect (Kruskal-Wallis test) are shown above the corresponding boxes. Symbols for significant differences in *post hoc* pairwise comparisons: ^∗^*p* < 0.05; ^∗∗^*p <* 0.01; ^∗∗∗^*p <* 0.001 for *Hq*-Sed vs. WT-Sed, and *Hq*-Ex vs.WT-Ex. ATP5α, ATP synthase subunit alpha; Core 2, ubiquinol-cytochrome C reductase core protein 2; COXI, cytochrome c oxidase subunit I; CS, citrate synthase; HSP60, heat shock protein 60; NDUFA9, NADH:ubiquinone oxidoreductase subunit A9; NDUFB8, NADH:ubiquinone oxidoreductase subunit B8; NDUFS1, NADH:ubiquinone oxidoreductase subunit S1; NRF1, nuclear respiratory factor 1; SDHB, succinate dehydrogenase complex iron sulfur subunit B; VDAC1, voltage-dependent anion-selective channel protein 1.

Analysis of representative subunits of OXPHOS complexes in the cerebellum detected by western blotting are shown in Figure 1A. A significant group effect was found for the protein level of subunits of the three functional modules of complex I – NADH:ubiquinone oxidoreductase subunit B8 (NDUFB8, *p* < 0.0001), A9 (NDUFA9, *p* < 0.0001) and the core subunit 1 (NDUFS1, *p* = 0.0005) – and the complex IV subunit cytochrome oxidase subunit 1 (COXI, *p* = 0.0163), with levels in all cases lower in *Hq* mice than in WT mice irrespective of training status with the exception of COXI, in which *post hoc* differences were only observed between the two sedentary groups, and without differences between exercised and sedentary animals within genotypes. A significant group effect was also found for ATP synthase subunit α (ATP5α, *p* = 0.0013) with levels higher in exercised *Hq* mice than in exercised WT mice, and without differences between exercised or sedentary animals within genotypes.

Regarding mitochondrial content indicators, we found a significant group effect for voltage-dependent anion-selective channel protein 1 (VDAC1, *p* < 0.0001), heat shock protein 60 (HSP60, *p* = 0.0013) and nuclear respiratory factor 1 (NRF1, *p* = 0.0029), with lower levels of NRF1 but higher levels of VDAC1 and HSP60 in *Hq* relative to WT mice irrespective of training status, and without differences between exercised or sedentary animals within genotypes (Figure 1B). No significant group effect was found for citrate synthase (CS).

**FIGURE 2:**
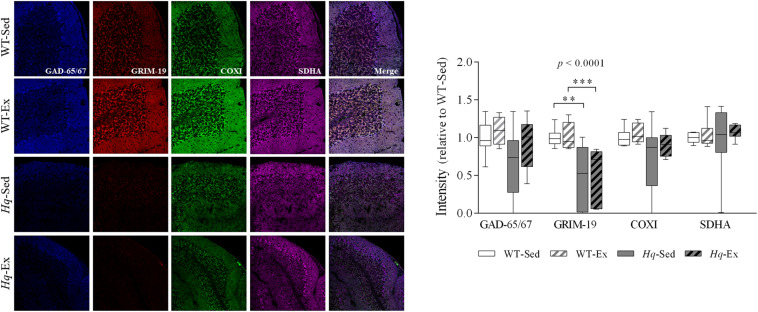
Immunohistochemistry analysis of respiratory complexes and Purkinje neuron markers. Immunodetection of markers of Purkinje cells, GAD-65/67 (blue); respiratory chain complex I, GRIM-19 (red); complex IV, COXI (green); complex II, SDHA (violet); and merged images in slices of cerebellum of wild-type sedentary (WT-Sed, *n* = 10) and exercise-trained (WT-Ex, *n* = 9) mice, and *Hq* sedentary (*Hq-*Sed, *n* = 10), and exercise-trained (*Hq*-Ex, *n* = 8) mice. Quantitative analysis of the signal intensities: data (median, interquartile range, and min and max values) are expressed relative to the intensity of the WT-Sed group. Significant *p-*values for group effect (Kruskal-Wallis test) are shown above the corresponding boxes. Symbols for significant differences in *post hoc* pairwise comparisons: ^∗∗^*p* < 0.01; ^∗∗∗^*p* < 0.001 for *Hq*-Sed *vs* WT-Sed, and *Hq*-Ex vs. WT-Ex. Abbreviations: COXI, cytochrome c oxidase subunit I; GAD-65/67, glutamate descarboxilase 65/67; GRIM-19, gene associated with retinoic and interferon-induced mortality 19 protein; SDHA, succinate dehydrogenase complex iron sulfur subunit A. Analyses were performed in 1,000 cells (*i.e.*, 187 cells for 7 WT-Sed animals; 168 cells for 5 WT-Ex animals; 293 cells for 6 *Hq*-Sed 6 animals, and 352 cells for 5 *Hq*-Ex animals).

To specifically assess complex I and IV deficiency in cerebellar Purkinje cells, we performed quadruple immunohistochemistry against gene associated with retinoic and interferon-induced mortality 19 protein (Grim-19) and COXI in tissue sections, using anti-glutamic acid decarboxylase 65/67 (GAD-65/67), and succinate dehydrogenase complex subunit A (SDHA) antibodies as markers of Purkinje cell and mitochondria, respectively (Figure 2). A significant group effect (*p* < 0.0001) was found for Grim-19, with levels lower in *Hq* mice than in WT mice irrespective of training status, and without differences between exercised or sedentary animals within genotypes. No group effect was found for the remaining variables (COXI, GAD-65/67, or SDHA). Indeed, the intensity staining of SDHA was similar between all the experimental groups, suggesting the absence of changes in mitochondrial content in the Purkinje cells due to AIF deficiency or exercise training.

### Neuron Loss

**FIGURE 3:**
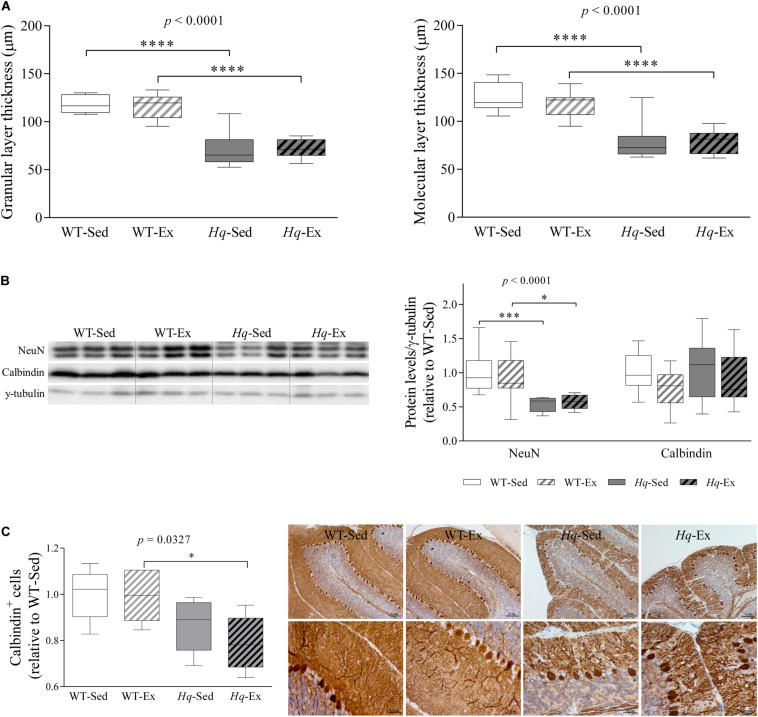
Cerebellar atrophy and neuronal loss. **(A)** Mean thickness of granular and molecular layers of cerebellar cortex. Analyses were performed in 9 WT-Sed, 8 WT-Ex, 11 *Hq*-Sed and 9 *Hq*-Ex animals, using 1 section per mouse. **(B)** Western blot analysis of NeuN and calbindin in cerebellum homogenates of wild-type sedentary (WT-Sed, *n* = 12) and exercise-trained (WT-Ex, *n* = 11) mice, and of *Hq* sedentary (*Hq*-Sed, *n* = 11) and exercise-trained (*Hq*-Ex, *n* = 10) mice. Densitometry analysis: protein levels were normalized to γ-tubulin levels; data (median, interquartile range, and min and max values) are expressed relative to levels in the WT-Sed group. **(C)** Immunohistochemistry analysis of calbindin in sagittal sections of cerebellum of representative WT-Sed, WT-Ex, *Hq*-Sed and *Hq*-Ex mice (10× and 40×) and quantitative analysis of calbindin-positive cells (Purkinje neurons) per mm in cerebellar cortex. Significant *p-*values for the group effect (Kruskal-Wallis test) are shown above the corresponding boxes. Symbols for significant differences in *post hoc* pairwise comparisons: ^∗^*p* < 0.05; ^∗∗∗^*p* < 0.001, ^*⁣*⁣**^p < 0.0001 for *Hq*-Sed vs. WT-Sed, and *Hq*-Ex vs. WT-Ex. Analyses were performed in 5 animals for each of the four experimental groups, 2–6 images per animal.

A significant group effect was found for the granular and molecular layers thickness (both *p* < 0.0001), with levels lower in *Hq* mice than in WT mice irrespective of training status, and without differences between exercised or sedentary animals within genotypes (Figure 3A). The loss of granular and Purkinje cells in the cerebellum of *Hq* mice was semi-quantified by western blotting using antibodies against the specific markers Fox-3 (also known as Rbfox3 or hexaribonucleotide binding protein-3 [NeuN]) and calbindin (Figure 3B). A significant group effect was found for NeuN (*p* < 0.0001), with levels lower in *Hq* mice than in WT mice irrespective of training status, and no significant group effect was found for calbindin. However, calbindin-positive cell counting in tissue sections demonstrated that Purkinje cells were significantly fewer in *Hq* mice than in WT mice, and without differences between exercised or sedentary animals within genotypes (*p* = 0.0327) (Figure 3C, left panel). Histological staining also demonstrated a marked reduction in the complexity of Purkinje cell dendrites in *Hq* mice when compared with WT mice (Figure 3C, right panel), a phenomenon that exercise training did not seem to attenuate.

Analysis of the pre-intervention group also showed lower NeuN levels in the cerebellum of *Hq* mice when compared with their healthy age-matched controls (1.0**±** 0.16 and 0.77 ± 0.06, WT and *Hq*, respectively, *p* = 0.048). Together with the cerebellar atrophy also observed in *Hq* mice (data not shown), this finding indicates that granule cell degeneration had already begun before the exercise intervention had started. This suggests that once the neurodegenerative process is initiated, the exercise intervention is unable to delay granular cell death or the subsequent loss of dendrites of Purkinje cells — with both phenomena evident at the end of the intervention period in the two *Hq* groups.

### Apoptosis

**FIGURE 4:**
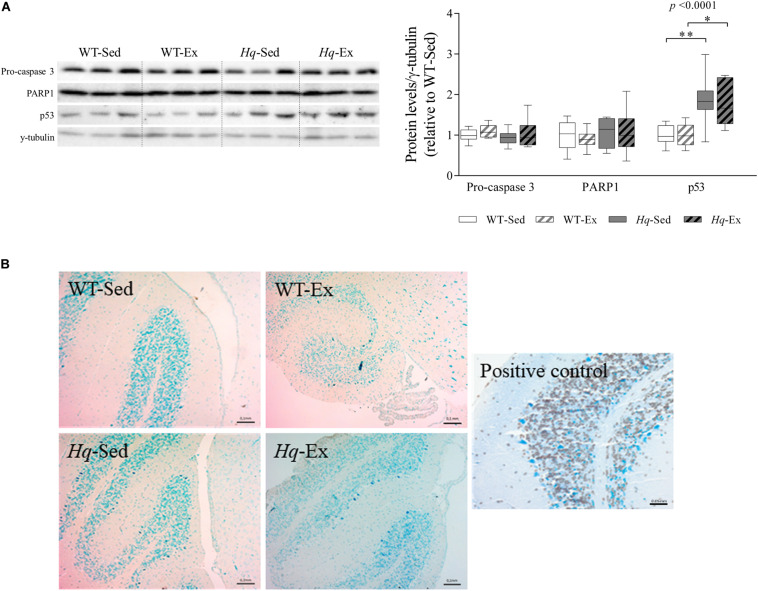
Apoptosis markers. **(A)** Western blot analysis of pro-caspase 3, poly [ADP-ribose] polymerase 1 (PARP1) and p53 in cerebellum homogenates of wild-type sedentary (WT-Sed, *n* = 12) and exercise-trained (WT-Ex, *n* = 11) mice, and of *Hq* sedentary (*Hq*-Sed, *n* = 11) and exercise-trained (*Hq*-Ex, *n* = 10) mice. Densitometry analysis: protein levels were normalized to γ-tubulin levels; data (median, interquartile range, and min and max values) are expressed relative to levels in the WT-Sed group. Significant *p-*values for the group effect (Kruskal-Wallis test) are shown above the corresponding boxes. Symbols for significant differences in *post hoc* pairwise comparisons: ^∗^*p* < 0.05; ^∗∗^*p* < 0.01 for *Hq*-Sed vs. WT-Sed, and *Hq*-Ex vs. WT-Ex. **(B)** Detection of apoptotic nuclei in cerebellar cortex by TUNEL assay, counterstained with hematoxylin (10×). DNase-treated samples were used as a positive control. Analyses were performed in 12 WT-Sed, 11 WT-Ex, 11 *Hq*-Sed and 9 *Hq*-Ex mice (1 section per mouse).

To assess whether neuron loss in *Hq* mice was due to higher levels of apoptosis, as previously reported (Klein et al., 2002), and to also examine the potential effects of the exercise intervention on this mechanism, we measured apoptosis effector protein abundance by western blotting (*i.e.*, pro-caspase 3 and active caspase 3, and the active form of poly ADP ribose polymerase 1 [PARP1]). No significant group effect was found for pro-caspase 3 or PARP1 (Figure 4A), and active caspase 3 was not detected in any experimental group. However, we found a significant group effect (*p* < 0.0001) for p53 – an alternative pathway to the canonical caspase-induction pathway that also induces apoptosis in response to cellular stressors (Stambolsky et al., 2006). Specifically, p53 levels were higher in *Hq* mice than in WT mice irrespective of training status, and without differences between exercised or sedentary animals within genotypes. Finally, we used the terminal deoxynucleotidyl transferase (TdT)-mediated dUTP nick-end labeling (TUNEL) staining to test whether p53-induced apoptosis was active, but we failed to detect any TUNEL-positive (apoptotic) cell in WT or *Hq* cerebellum tissue irrespective of their training status (Figure 4B). By contrast, when we studied the younger, pre-intervention *Hq* and WT mouse groups (those sacrificed at 6–8 weeks of age vs. 22 weeks on average for the four study groups), we detected apoptotic granule cells in the cerebellum of *Hq* animals but not in their WT referents (data not shown).

### Oxidative Stress Markers and Antioxidant Enzymes

**FIGURE 5:**
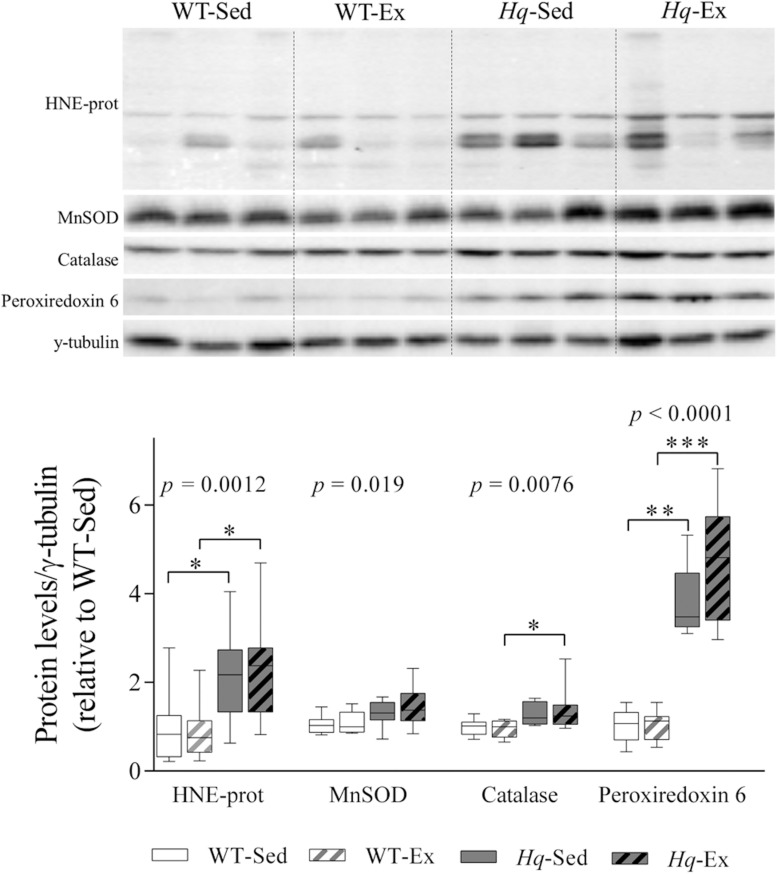
Oxidative stress marker and antioxidant enzymes. Western blot analysis of 4-hydroxynonenal-modified proteins (HNE-prot), manganese superoxide dismutase (MnSOD), catalase and peroxiredoxin 6, in cerebellum homogenates of wild-type sedentary (WT-Sed, *n* = 12) and exercise-trained (WT-Ex, *n* = 11) mice, and of *Hq* sedentary (*Hq*-Sed, *n* = 11) and exercise-trained (*Hq*-Ex, *n* = 10) mice. Densitometry analysis: protein levels were normalized to γ-tubulin levels; data (median, interquartile range, and min and max values) are expressed relative to levels in the WT-Sed group. Significant *p-*values for the group effect (Kruskal-Wallis test) are shown above the corresponding boxes. Symbols for significant differences in *post hoc* pairwise comparisons: ^∗^*p* < 0.05; ^∗∗^*p* < 0.01; ^∗∗∗^*p* < 0.001 for *Hq*-Sed vs. WT-Sed, and *Hq*-Ex vs. WT-Ex.

To determine whether exercise training could attenuate the oxidative stress previously described in the *Hq* mouse model (Klein et al., 2002), we measured the levels of proteins modified by the lipid peroxide hydroxynonenal (HNE-prot), and of three antioxidant enzymes (mitochondrial superoxide dismutase [MnSOD], catalase, and peroxiredoxin 6) by western blotting (Figure 5). A significant group effect was found for HNE-prot (*p* = 0.0012), MnSOD (*p* = 0.019), catalase (*p* = 0.0076) and peroxiredoxin 6 (*p* < 0.0001), with significant *post hoc* differences for all these variables with the exception of MnSOD, with overall higher levels in *Hq* relative to WT mice irrespective of training status, and without differences between exercised or sedentary animals within genotypes.

### Astroglial and Microglial Activation

**FIGURE 6:**
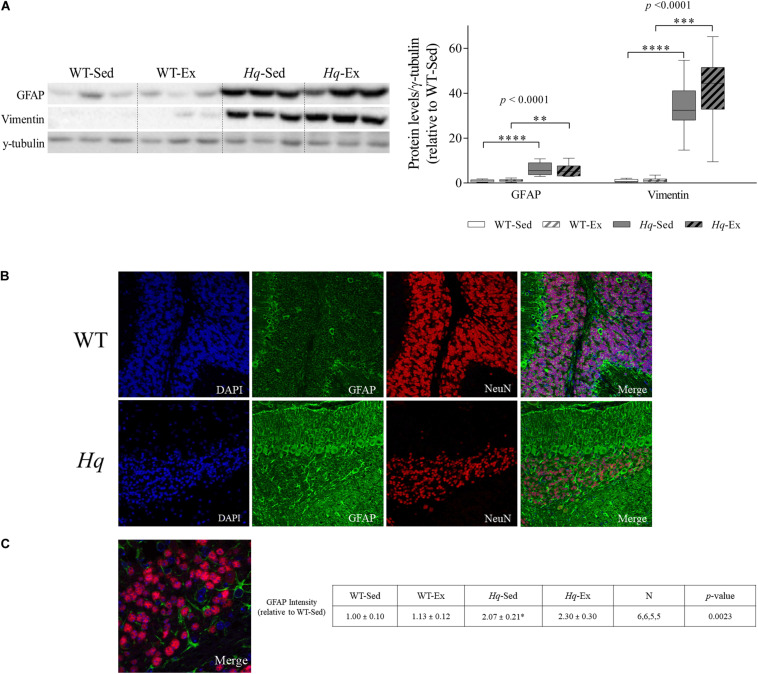
Astrogliosis markers. **(A)** Western blot analysis of glial fibrillary acidic protein (GFAP) and vimentin in cerebellum homogenates of wild-type sedentary (WT-Sed, *n* = 12) and exercise-trained (WT-Ex, *n* = 11) mice, and of *Hq* sedentary (*Hq*-Sed, *n* = 11) and exercise-trained (*Hq*-Ex, *n* = 10) mice. Densitometry analysis: protein levels were normalized to γ-tubulin levels; data (median, interquartile range, and min and max values) are expressed relative to levels in the WT-Sed group. Significant *p-*values for the group effect (Kruskal-Wallis test) are shown above the corresponding bars. Symbols for significant differences in *post hoc* pairwise comparisons: ^∗^*p* < 0.05; ^∗∗^*p* < 0.01; ^∗∗∗^*p* < 0.001; ^*⁣*⁣**^p < 0.0001 for *Hq*-Sed vs. WT-Sed, and *Hq*-Ex vs. WT-Ex. **(B)** Immunodetection of GFAP (green) and NeuN (red), cell nuclei (blue) and merged images in representative sedentary WT and *Hq* mice (10×). Inmunofluorescence semi-quantitation was performed in 6 WT-Sed, 6 WT-Ex, 5 *Hq*-Sed and 5 *Hq*-Ex animals (1 section per mouse). Only representative WT-Sed and *Hq*-Sed images are shown for simplicity purposes, due to absence of training-induced effect. **(C)** Higher magnification images showing direct interaction between GFAP-positive astrocytes (green) and granule neurons (red) in a representative sedentary *Hq* mouse (63×).

To assess the effects of the exercise training on neuroinflammation, we analyzed the intermediate filament proteins glial fibrillary acidic protein (GFAP) and vimentin, indicative of astroglial activation, that were considerably more abundant in the cerebellum of *Hq* mice, finding a significant group effect for both proteins (*p* < 0.0001), with higher levels in *Hq* mice than in WT mice irrespective of training status and without differences between exercised or sedentary animals within genotypes (Figure 6A). We also analyzed GFAP by immunofluorescence, finding higher mean fluorescence levels (which are indicators of astroglial activation) in the cerebellar cortex of both *Hq* groups compared to WT mice (*p* = 0.0023), with significant *post hoc* differences for *Hq*-Sed vs. WT-Sed (Figures 6B,C). Closer inspection of higher magnification images showed granule cell engulfment by astrocytic processes in *Hq* mice, a phenomenon much less frequent in WT cerebellar tissue (Figure 6C).

**FIGURE 7:**
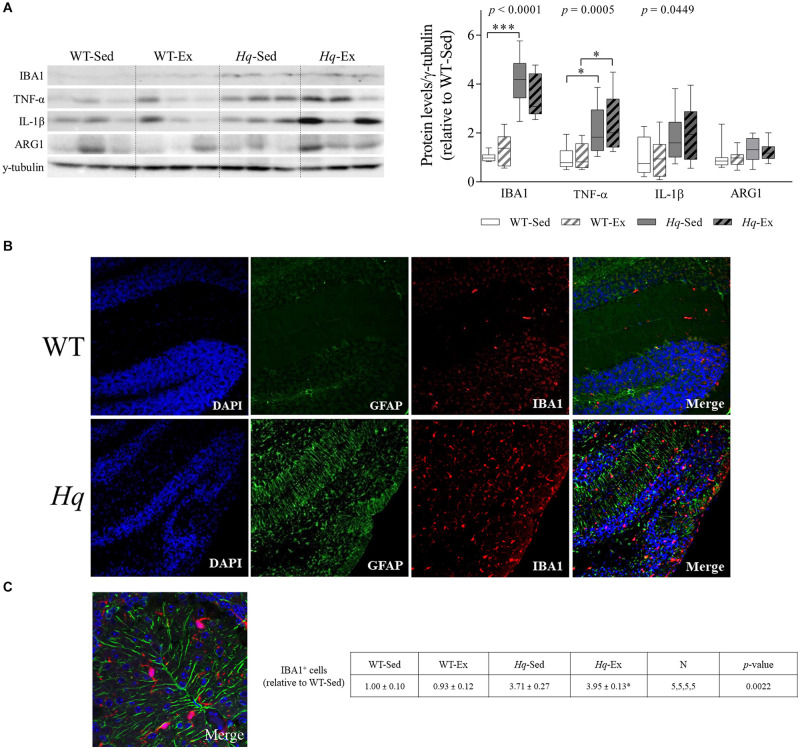
Microglial activation and polarization markers. **(A)** Western blot analysis of ionized calcium binding adapter molecule 1(IBA1), tumor necrosis factor α (TNF-α), interleukin 1 β (IL-1β), and arginase 1 (ARG1) in cerebellum homogenates of wild-type sedentary (WT-Sed, *n* = 12) and exercise-trained (WT-Ex, *n* = 11) mice, and of *Hq* sedentary (*Hq*-Sed, *n* = 11) and exercise-trained (*Hq*-Ex, *n* = 10) mice. Densitometry analysis: protein levels were normalized to γ-tubulin levels; data (median, interquartile range, and min and max values) are expressed relative to levels in the WT-Sed group. Significant *p-*values for the group effect (Kruskal-Wallis test) are shown above the corresponding boxes. Symbols for significant differences in *post hoc* pairwise comparisons: ^∗^*p* < 0.05; ^∗∗∗^*p* < 0.001 for *Hq*-Sed vs. WT-Sed, and *Hq*-Ex vs. WT-Ex. **(B)** Immunofluorescence analysis of cerebellar cortex for detection of GFAP (green), and IBA1 (red), and nuclei (blue), and merged images in representative sedentary WT and *Hq* mice (10×). Analyses were performed in 5 animals for each experimental group, 1 slide per animal. Only representative WT-Sed and *Hq*-Sed images are shown for simplicity purposes, due to absence of training-induced effect. **(C)** Higher magnification image of a representative sedentary *Hq* mouse showing activated microglial cells in both granule and molecular layers (63×).

To further assess whether microglial cell activation occurred in the cerebellum of *Hq* mice, we analyzed the levels of ionized calcium binding adaptor molecule 1 (IBA1) by western blotting in total cerebellum homogenates. A significant group effect was found for this microglial marker (Figure 7A) (*p* < 0.0001), with levels higher in sedentary *Hq* mice than in sedentary WT mice, and without differences between exercised or sedentary animals within genotypes. These results correlated well with the immunofluorescence analysis of this marker in tissue sections, with evident IBA1-positive cells in both granular and molecular layers (Figures 7B,C). IBA1-positive cells per mm^2^ were more abundant in both *Hq* mice groups than in WT mice irrespective of training status (Figure 7C) (*p* = 0.0022). To evaluate the pro- (M1-like) or anti-inflammatory (M2-like) polarization of activated microglial cells, we determined the levels of the pro-inflammatory cytokines tumor necrosis factor (TNF)-α and interleukin (IL)-1β, as markers of M1-like cerebellar microglia, and arginase 1 (ARG1) as an M2 marker (Figure 7A). The results showed a significant group effect for TNF-α (*p* = 0.0005) and IL-1β (*p* = 0.0449), with levels of the former higher in *Hq* mice than in WT mice irrespective of training status, and without differences between exercised or sedentary animals within genotypes. Our results thus reflect astrocyte and microglial activation and polarization towards a pro-inflammatory or M1-like phenotype in the *Hq* mouse cerebellum, a phenomenon that the exercise intervention failed to attenuate.

### Exercise Training Effects on Brain Variables

To assess if the training intervention was able to induce any significant effect on other regions of the CNS, we analyzed several variables related to OXPHOS system, oxidative stress and neuroinflammation in brain total homogenates by western blot (Table 1). In brain, a significant group effect was found for the levels of NDUFB8, NDUFS1 and COXI (*p* < 0.0001, *p* = 0.0028, and *p* = 0.0044, respectively), with *post hoc* differences observed for NDUFB8 in both *Hq* groups vs. their respective WT controls, and for the *Hq*-Ex vs. WT-Ex for COXI, and without differences between exercised or sedentary animals within genotypes. We did not observe any group effect neither in the oxidative stress marker HNE-prot, nor in the antioxidant enzymes levels (Table 1). On the contrary, a significant group effect was detected for GFAP, that was higher in both *Hq* mice groups, with *post hoc* differences only between *Hq*-Sed and WT-Sed animals (*p* < 0.0001).

**TABLE 1:** Brain variables.

	**WT-Sed**	**WT-Ex**	***Hq*-Sed**	***Hq*-Ex**	**N with data**	***p*-value for group effect**
NDUFB8	1.00 ± 0.14	1.05 ± 0.14	0.26 ± 0.04**	0.23 ± 0.05***	12,11,11,10	<0.0001
NDUFA9	1.00 ± 0.12	1.14 ± 0.07	1.01 ± 0.09	0.79 ± 0.05	12,11,11,10	Ns
NDUFS1	1.00 ± 0.07	1.23 ± 0.15	0.80 ± 0.09	0.69 ± 0.06	12,11,11,10	0.0028
SDHB	1.00 ± 0.11	1.24 ± 0.17	1.13 ± 0.15	1.13 ± 0.15	12,11,11,10	Ns
Core2	1.00 ± 0.05	1.13 ± 0.12	0.94 ± 0.911	0.92 ± 0.11	12,11,11,10	Ns
COXI	1.00 ± 0.05	1.14 ± 0.12	0.87 ± 0.10	0.75 ± 0.08*	12,11,11,10	0.0044
ATP5α	1.00 ± 0.05	1.16 ± 0.13	0.83 ± 0.13	0.92 ± 0.09	12,11,11,10	Ns
CS	1.00 ± 0.06	0.90 ± 0.08	0.86 ± 0.06	0.85 ± 0.06	12,11,11,10	Ns
HNE-Prot	1.00 ± 0.13	0.90 ± 0.22	1.04 ± 0.15	0.69 ± 0.15	8,9,8,8	Ns
MnSOD	1.00 ± 0.07	0.97 ± 0.08	1.08 ± 0.05	1.12 ± 0.07	11,11,11,10	Ns
Catalase	1.00 ± 0.04	0.93 ± 0.04	0.97 ± 0.03	1.03 ± 0.5	12,11,11,10	Ns
PRDX6	1.00 ± 0.13	0.64 ± 0.08	1.14 ± 0.21	1.00 ± 0.14	12,10,11,10	Ns
GFAP	1.00 ± 0.22	1.35 ± 0.39	4.01 ± 0.36***	3.38 ± 0.57	12,11,11,10	<0.0001

Data are expressed as mean ± standard error of the mean. The Kruskal-Wallis test was detected whether a group effect existed, with statistical significance set at 0.05 and the Tukey test applied for post hoc pairwise comparisons. Symbols for *post hoc* comparisons: *p < 0.05, **p < 0.01, ***p < 0.001 for *Hq*-Sed *vs* WT-Sed, and *Hq*-Ex *vs* WT-Ex. Ns, not significant; WT-Sed, WT sedentary; WT-Ex, exercise-trained WT; *Hq*-Sed, sedentary *Hq*; *Hq*-Ex, exercise-trained *Hq*.

**FIGURE 8:**
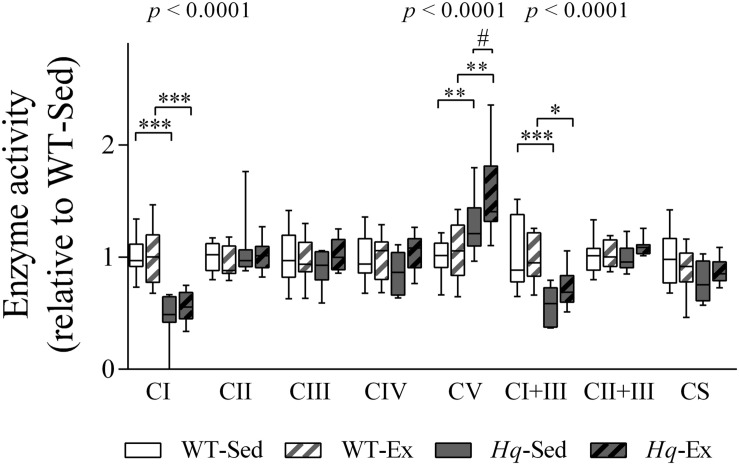
OXPHOS enzymatic activities in brain homogenates. Enzyme activities in total brain homogenates of wild-type sedentary (WT-Sed, *n* = 12), and exercise-trained (WT-Ex, *n* = 11) mice, and of *Hq* sedentary (*Hq*-Sed, *n* = 11) and exercise-trained (*Hq*-Ex, *n* = 10) mice. Data (median, interquartile range, and min and max values) are expressed relative to levels in the WT-Sed group. Significant *p-*values for the group effect (Kruskal-Wallis test) are shown above the corresponding boxes. Symbols for significant differences in *post hoc* pairwise comparisons: ^∗^*p* < 0.05; ^∗∗^*p* < 0.01; ^∗∗∗^*p* < 0.001 for *Hq*-Sed vs. WT-Sed, and *Hq*-Ex vs. WT-Ex; ^#^*p* < 0.05 for *Hq*-Ex vs. *Hq*-Sed. Abbreviations: CI, II, III, IV, and V, respiratory complexes I, II, III, IV, and V; CI+III, activity of complex I coupled to complex III; CII+III, activity of complex II coupled to complex III; CS, citrate synthase.

To determine if the training effect was able to promote functional changes in the OXPHOS system we measured their enzyme activities in the brain homogenates (Figure 8). According to the lower level of some complex I subunits in brain, we detected a significant group effect for the activities of complex I, and complex I coupled to complex III (*p* < 0.0001, both variables), with lower activities in both *Hq* groups in comparison with their respective WT controls, and without differences between exercised or sedentary animals within genotypes. Also, a significant effect was observed for complex V activity (*p* < 0.0001), that, in turn, was higher in both *Hq* mice groups in comparison with WT animals, and *post hoc* analysis also revealed differences between sedentary and trained *Hq* mice.

## Discussion

The main finding of the present study was that despite the strong improvements in aerobic capacity and muscle strength recently reported by us in the same animal model (Fiuza-Luces et al., 2019), the exercise training intervention failed to counteract the neurodegeneration observed in the cerebellum – as well as in brain – of *Hq* mice.

Physical exercise has a potential neuroprotective effect, especially at the level of the hippocampus (Valenzuela et al., 2020). First, it promotes neurogenesis *via* increases in exercise-induced metabolic factors such as lactate and ketone bodies, which are able to cross the blood-brain barrier (BBB). Voluntary aerobic exercise leads to the accumulation of lactate in the hippocampus, where it promotes an improvement of cognitive function (learning and memory) *via* increased expression of brain-derived neurotrophic factor (BDNF) (El Hayek et al., 2019). Prolonged aerobic exercise also induces the production of ketone bodies (β-hydroxybutyrate and acetoacetate), which stimulate the production of BDNF at the brain level (Sleiman et al., 2016; Hu et al., 2018). In addition, muscle contractions induce the release of molecules (mostly small peptides) into the bloodstream known as ‘myokines’, such as cathepsin-B (Moon et al., 2016) and irisin (Wrann et al., 2013), which can cross the BBB to induce neuroprotection. Previous studies have also found a beneficial effect of exercise training on neuroinflammation. Physical exercise has been indeed reported to promote a phenotypic conversion of M1 to M2 microglia along with an increased and reduced expression of anti- and pro-inflammatory cytokines, respectively, in different rodent models of neurodegeneration (*e.g*., aging, Alzheimer’s disease) (Kohman et al., 2013; He et al., 2017; Jiang et al., 2017; Lu et al., 2017). In addition, physical exercise seems to enhance antioxidant capacity in the brain, as confirmed in healthy rodent models of different ages (Camiletti-Moirón et al., 2013).

Consistent with the hypothesis of a neuroprotective effect of exercise, previous findings in the *Mutator* mouse model of MD and in other rodent models of neurodegeneration have demonstrated aerobic training-induced mitigation of mitochondrial dysfunction in the CNS (Safdar et al., 2011; Lai et al., 2019), including oxidative stress, neuron loss, neuroinflammation or protein deregulation (Clark-Matott et al., 2015; Do et al., 2018; Ross et al., 2019). The fact that our intervention started when the first signs of ataxia were evident and neuronal cell death had already occurred might explain, at least partly, why we did not find significant improvements. For example, in the studies performed with the *Mutator* mouse (a model of progeroid aging due to a mutation in mitochondrial DNA polymerase), whose age of symptoms (not directly related to the CNS) onset is usually 25 weeks (Trifunovic et al., 2004), exercise training initiating before symptom onset (*i.e.*, at 3 months of age) was able to normalize deficits in neurotransmitters as well as levels of carnitine, oxidative stress, and protein expression (Safdar et al., 2011; Clark-Matott et al., 2015; Ross et al., 2019). Moreover, in those studies the duration of the exercise intervention was 5–6 months, which is more than twice the duration of the present intervention. Likewise, in mouse models of Alzheimer (Do et al., 2018) and Parkinson (Lai et al., 2019) disease, in which exercise training-induced attenuation of neuroinflammation, cell death, and oxidative function has been reported, the training programs started before the onset of symptoms, and in the Parkinson disease study the training program was in fact longer that the one we applied here (Lai et al., 2019). Thus, an early start of the training intervention, as well as long-term protocols, could be key factors to promote neuroprotection in mouse models of neurodegeneration. This being said, early exercise interventions are difficult to implement in patients with MD, as the time of symptom onset usually precedes that of diagnosis. As such, our exercise intervention mimics real-world clinical scenarios. In this context, it seems that exercise training in patients diagnosed with MD is likely to induce significant benefits in cardiorespiratory and muscle fitness, which is clinically relevant because both variables are markers of cardiometabolic health (Fiuza-Luces et al., 2018b), but will not attenuate cerebellar degeneration.

Exercise training can induce motor skill learning through the promotion of synaptic plasticity and remodeling in those areas involved in the activity. Accordingly, long-term programs of physiotherapy designed to promote neural plasticity, as well as new video game-based therapy, improve ataxia symptoms (Aranca et al., 2016; Wang et al., 2018). In the present study, we observed the same extent of atrophy in the dendritic tree of Purkinje cells in sedentary and trained *Hq* mice, demonstrating that exercise was unable to protect pre-existing synapses. Beyond the aforementioned issue related to the age at which the intervention started, the fact that the activity program we used was based more on unskilled motor movements (running on the treadmill, or resistance training) might also have contributed to the lack of intervention effects at the cerebellar level. However, we believe it is important to assess the effects of continuous muscle work (*i.e.*, aerobic and resistance exercises), as muscle contractions are known to elicit a neuroprotective milieu given the aforementioned muscle-brain crosstalk.

Although exercise training did not elicit any adaptive response in the cerebellum of *Hq* mice, this intervention induced higher complex V activity in the brain of trained *Hq* mice in comparison with their sedentary *Hq* counterparts, activity that was higher in both *Hq* groups than in WT animals. In the present study the activity of complex V was measured as ATPase (Kirby et al., 2007), therefore, it is tempting to speculate that in the brain of *Hq* mice the higher reverse complex V activity could be consuming glycolysis-derived ATP to sustain mitochondrial membrane potential, a key parameter for cell viability, that could not be maintained by the activity of respiratory chain, due to complex I deficiency. In fact, this compensatory mechanism has been reported formerly in isolated nerve terminals (Chinopoulos and Adam-Vizi, 2010), and our results indicate that it operates to a higher extent in the trained *Hq* mice. Previous studies in *Hq* mice have not reported changes in complex V activity (Vahsen et al., 2004; Bénit et al., 2008), probably due to methodological differences (*i.e.*, in the enzyme assay performed, the portion of the nervous system, or the cellular fraction analyzed). Further studies to assess the role of the reverse activity of complex V in the *Hq* mouse nervous tissue and its response to exercise training are needed.

Interventions other than exercise have been proposed as potentially beneficial in *Hq* mice. For instance, a mitochondrial redox compound (methylene blue) has been recently reported to attenuate oxidative stress and prevent retinal degeneration in this mouse model (Mekala et al., 2019). Overexpression of neuroglobulin – a molecule that exerts neuroprotective effects through modulating respiratory chain function – has been reported to prevent the neurodegeneration (*i.e.*, retinal ganglion cell loss, optic nerve atrophy) that would otherwise occur in *Hq* mice (Lechauve et al., 2014). In addition, *Hq* mice fed with a high-fat diet from weaning (1 month) have been reported to present with an attenuation of several disease-related markers (*e.g.*, phospholipid levels in cerebellum membranes, cerebellum weight, and rotarod performance) (Schiff et al., 2011). Future studies should confirm whether physical exercise protocols different to the one applied here or conducting the exercise intervention before symptoms appear could not only improve physical performance as we recently showed (Fiuza-Luces et al., 2019), but also prevent neurodegeneration.

There are some limitations in our study. The time course of the disease in the *Hq* mouse model precludes the possibility of implementing the training program before symptom onset. Indeed, molecular symptoms appear very early as demonstrated in pre-intervention *Hq* mice. The severity of the symptoms in this model also limits application of high exercise intensities, with the training workloads used in our study being possibly below the threshold needed to maximize biological adaptations. On the other hand, the potential stress situation associated with forced (treadmill) exercise in mice might have attenuated the potential neuroprotective effects of exercise, as opposed to the more ‘natural’ or ‘spontaneous’ voluntary wheel training model. We might have also failed to detect subtle positive training-induced effects due to technical limitations, such as the use of conventional cell counting or cerebellar layer thickness determination instead of the more accurate stereological methodology. In turn, there are some major strengths in our design, particularly the fact that the study was well controlled and that we assessed for the first time the effects of a combined training program including resistance training, an exercise modality that has not been previously tested in mouse models of MD.

In summary, a combined training program (aerobic and resistance exercises) initiated when neurological symptoms and neuron death are already apparent (*i.e.*, ∼3 months) is unlikely to promote a significant neuroprotection in a mouse model of MD, and reveals the difficulty of treating some severe conditions based only on lifestyle interventions.

## Materials and Methods

### Animals

All experimental protocols received Institutional Review Board approval (*Hospital 12 de Octubre*, project number PROEX111/15) and were conducted in accordance with ARRIVE guidelines and with European (European convention ETS 123) and Spanish (32/2007 and R.D. 1201/2005) laws on animal protection in research. Hemizygous (*n* = 21, *Hq*/Y hereafter named ‘*Hq*’, B6CBACa A^w–J^/A-Aifm1^Hq^/J) and WT male mice of the same strain (all 6–8 weeks old) were purchased from The Jackson Laboratory (Bar Harbor, ME, United States). Mice were housed in the animal facility of *Hospital 12 de Octubre* in controlled conditions of temperature (21°C), humidity (60%) and ventilation, with 12-h light/dark cycles and with *ad libitum* access to food and water.

### Study Design

Study variables were measured after training (*i.e.*, when animals were 24–26 weeks old). The animals and exercise training protocols have been described in detail in a recent study by our group (Fiuza-Luces et al., 2019). Briefly, *Hq* and WT mice were evaluated from 8 weeks of age until the first signs of ataxia appeared in the former. To ensure that all *Hq* mice presented with the same degree of disease affectation at the moment of their allocation to the exercise or control groups (see below), the locomotion ability of each animal was assessed by two independent examiners with the mouse walking on a treadmill and by scoring walking gait balance, ability to walk straight, and alterations in hind limb gait (Fiuza-Luces et al., 2019). Cerebellar ataxia was also tested using the rotarod device on alternative days during the evaluation phase. Data obtained from WT animals during the same period were used as a reference for absence of locomotor and coordination alterations. When according to the scores obtained in the evaluation the first signs of ataxia were evident, each *Hq* mouse and an age-paired WT animal performed an incremental treadmill test (after prior familiarization) as previously described (Fiuza-Luces et al., 2013). Mice were then paired-matched based on their aerobic capacity and assigned to an exercise (Ex) group, subjected to an 8-week exercise training program [WT-Ex [*n* = 11] and *Hq*-Ex (*n* = 10)], or to a control (non-exercise) sedentary (Sed) group, allowed to freely move in the cage but not performing the program [WT-Sed (*n* = 12) and *Hq*-Sed (*n* = 11)]. An additional ‘pre-intervention’ group of WT (*n* = 5) and *Hq* (*n* = 4) mice was sacrificed before the first signs of ataxia were evident (at 6–8 weeks of age).

### Exercise Intervention

Exercise training consisted of five sessions per week (Monday–Friday; session duration, 40–60 min), each including aerobic treadmill running, coordination (rotarod), and resistance or ‘strength’ (horizontal screen, two-limb hanging) exercises, as described (Fiuza-Luces et al., 2019). Before and after the period corresponding to the exercise training program, all the mice performed an exercise treadmill test, and a rotarod and a grip strength test to assess endurance capacity, coordination and muscular strength, respectively (Fiuza-Luces et al., 2019).

Regarding aerobic exercise, the duration, treadmill speed, and inclination were gradually increased during the 8-week program following an interval training protocol previously described with minor modifications (Fiuza-Luces et al., 2013). Thus, the relative workload was progressively and individually (*i.e.*, based on the baseline treadmill assessment test) risen for each mouse in the Ex groups, increasing from 20 min at 35% of the maximal velocity in the treadmill test and 0% gradient at the start of the program to 40 min at 65–70% of maximal velocity and 15% gradient in the last sessions. Resistance training included horizontal screen exercise, hanging with two limbs exercise and rotarod exercise. The time and/or repetitions of horizontal screen and hanging exercises were gradually increased during the training program. For the rotarod exercise animals walked the rotating rod at increasing speed from 4 to 40 rpm over a 300 s period, and after the first fall, the number of rpm was recorded, and the mouse exercised at this speed during 5 min (Fiuza-Luces et al., 2019).

Finally, 48 h after the last exercise capacity test, mice were sacrificed by cervical dislocation, and brain and cerebellum were dissected and processed.

### Tissue Processing

The left hemisphere of the brain and cerebellum, respectively, were immediately snap-frozen in liquid nitrogen and stored at 80°C for biochemical analysis. The right hemispheres (midsagittal section) were fixed in 4% paraformaldehyde and paraffin-embedded in blocks for histology (5-μm sections). Frozen tissues were processed to obtain total tissue homogenates with ice-cold RIPA buffer (50 mM Tris–HCl pH 7.4, 1% NP-40, 0.5% Na-deoxycholate, 1% SDS, 150 mM NaCl, 2 mM EDTA) plus protease and phosphatase inhibitors (Roche Diagnostics Corp., Indianapolis, IN, United States) using a Potter homogenizer. Protein concentration was determined with the BCA Assay Kit (Pierce, Thermo Scientific, Waltham, MA, United States).

### Western Blotting

Protein levels of respiratory chain complex subunits, antioxidant enzymes, markers of oxidative stress and proteins involved in glial activation and neuronal transmission were determined by western blotting. Briefly, samples of total homogenates were loaded onto SDS-PAGE gels (7.5 to 15%). Resolved proteins were transferred to PVDF membranes, blocked with 5% skimmed milk or bovine serum albumin, incubated with primary (Supplementary Table 1) and HRP-conjugated secondary antibodies, and finally developed with the ECL Prime Western Blotting Detection Reagent (Amersham GE Healthcare, Little Chalfont, United Kingdom). Densitometry was performed with ImageJ (National Institutes of Health, Bethesda, MD, United States).

### Immunohistochemistry and Immunofluorescence

Paraffin embedding, sectioning and hematoxylin-eosin staining for measurement of cerebellar layer thickness were performed according to standard protocols. Immunohistochemistry staining with diaminobenzidine (DAB) was used to count calbindin-positive Purkinje cells. Carazzi hematoxylin (Panreac, Darmstadt, Germany) was used as a counterstain, and slides were mounted with Limonene mounting medium (Abcam, Cambridge, United Kingdom). Immunofluorescence in paraffin-embedded tissues slides was performed for multiple protein staining. After deparaffinization and rehydration, antigen retrieval was performed in citrate buffer pH 6.0 or Tris-EDTA buffer pH 9.0, at 95–100° C for 20 min. Slides were permeabilized and blocked in 2% BSA, 0.1% Triton X-100 in Tris-buffered saline (TBS) solution, incubated with the corresponding primary antibodies, washed and incubated with fluorescent secondary antibodies. Slides were finally incubated with 4’,6-diamidino-2-phenylindole (DAPI) and mounted with Dako Fluorescent Mounting Medium (Agilent Technologies Inc., Santa Clara, CA). Visualization of slides and image processing were performed with a Zeiss LSM510 META confocal scanning microscope (Carl Zeiss MicroImaging GmbH, Jena, Germany).

Cerebellar layers thickness and calbindin and IBA1-positive cells were counted in cerebellum sections using the Image J tools to count and measure objects, calibrating the images according to the magnification of the objective. To determine cerebellar layer thickness, the minimum distance between the inner and outer limit of each layer was determined every 0.1 mm of the lobule, and the mean value was taken as the lobule layer thickness. The number of calbindin-positive cells in each lobule was counted and corrected by the lobule outline measured in mm, with data expressed as number of positive cells per mm. IBA1 positive cells were counted in histological sections and expressed as number of cells per mm^2^.

Quadruple immunofluorescence was carried out in cerebellum sections (5 μm) using antibodies detecting subunits of OXPHOS complexes (Supplementary Table 1) according to previously described methods (Dobson et al., 2016). Briefly, after deparaffinization and rehydration of the sections, antigen retrieval was performed in 1 mM EDTA pH 8 for 40 min in a pressure cooker and sections were washed in distilled water. Sections were then blocked in TBS containing 1% Tween-20 (TBS-T) and 10% goat serum for 2 h at room temperature. After additional avidin-biotin blocking, the samples were incubated with primary antibodies diluted in 10% goat serum/TBS-T (monoclonal mouse antibodies against COXI, GRIM-19 and SDHA, and an anti-GAD-65/67 antibody, produced in rabbit) and incubated overnight at 4° C. After three washes with TBS-T, sections were incubated for 2 h at room temperature in 10% goat serum/TBS-T containing a biotinylated anti-rabbit antibody. After subsequent washes, sections were incubated with mouse IgG-specific secondary antibodies (Alexa Fluor 488, 546, and 647 nm) and streptavidin-405 nm in 10% goat serum/TBS-T. Finally, samples were washed again and mounted with ProLong Gold (Life Technologies, Carlsbad, CA, United States). To assess the mitochondrial defect in Purkinje cells, the tool ‘find objects’ of the Volocity software (Quorum Technologies Inc., Laughton, United Kingdom) was used to define a region of interest (ROI) surrounding each Purkinje cell. Every ROI was cropped and we determined the average signal intensity for each fluorophore. To assess non-specific background fluorescence, no primary control section was used to obtain average signal intensity levels at each fluorophore wavelength; we used the whole cell surface, and this value was fixed as background level.

### Respiratory Chain Complex Activity

Activities of respiratory chain complexes I–V were determined in cerebellum homogenates prepared in 225 mM mannitol, 75 mM sucrose, 0.1 mM ethylenediaminetetraacetic acid, and 10 mM Tris–HCl pH 7.4, according to standardized protocols for spectrophotometric assays (Medja et al., 2009). Between 20 and 60 μg of protein were used for respiratory chain complex measures. Citrate synthase (CS) activity was measured as previously described, after formation of 5-thio-2-nitrobenzoic acid in assay buffer containing 0.1% Triton X-100 and 20 μg of protein (Trounce et al., 1996). Complex V activity was assayed as F1-ATPase using 60 μg of protein as described elsewhere (Kirby et al., 2007).

### TUNEL Assay

The TUNEL assay was performed to detect apoptotic cells *in situ* in cerebellar cortex (Abcam, Cambridge, United Kingdom). Slides treated with 90U DNase enzyme (Sigma-Aldrich, Saint Louis, MO, United States) were used as a positive control.

### Statistical Analysis

All study variables were expressed as median, interquartile range, and minimum and maximum values in box and whisker plots. All variables were tested for normality of data distribution using the D’Agostino-Pearson test. Owing to the relatively small sample size and to the fact that almost 50% of the study variables did not follow a Gaussian (normal) distribution, the non-parametric equivalent of the one-factor (*i.e.*, group) ANOVA, the Kruskal-Wallis test, was used for all multiple group comparisons. When a significant ‘group effect’ was found the Tukey test was applied ‘*post hoc*’ for pairwise comparisons. Statistical significance was set at a *p*-value < 0.05 and all analyses were performed with GraphPad Prism v6 for Windows (GraphPad Software, San Diego, CA, United States).

## Data Availability Statement

The raw data supporting the conclusions of this article will be made available by the authors, without undue reservation.

## Ethics Statement

The animal study was reviewed and approved by Institutional Review Board (approval Hospital 12 de Octubre, project number PROEX111/15).

## Author Contributions

MFT: experimental procedures and drafting of the manuscript. PV: statistical analysis and drafting of the manuscript. CF-L and SL-M: experimental procedures. MAM and JA: critical review of the manuscript. DMT: critical review of the manuscript and direction of experimental procedures. AL: drafting of the manuscript. MM: study design, drafting of the manuscript, and direction. All authors revised the manuscript critically for important intellectual content, and approved the final version.

## Conflict of Interest

The authors declare that the research was conducted in the absence of any commercial or financial relationships that could be construed as a potential conflict of interest.

## References

[B1] Adhihetty P. J. Taivassalo T. Haller R. G. Walkinshaw D. R. Hood D. A. (2007). The effect of training on the expression of mitochondrial biogenesis- and apoptosis-related proteins in skeletal muscle of patients with mtDNA defects. *Am. J. Physiol. Endocrinol. Metab.* 293 672–680. 10.1152/ajpendo.00043.2007 17551003

[B2] Aranca T. V. Jones T. M. Shaw J. D. Staffetti J. S. Kuo S. Fogel B. L. (2016). Emerging therapies in Friedreich ’ s ataxia. *Neurodegener. Dis. Manag.* 6 49–65. 26782317 10.2217/nmt.15.73 PMC4768799

[B3] Ayvat E. Kilınç ÖO. Ayvat F. Sütçü G. Kılınç M. Aksoy S. (2018). The use of Goal Attainment Scaling (GAS) in the rehabilitation of ataxic patients. *Neurol. Sci.* 39 893–901. 10.1007/s10072-018-3304-7 29500687

[B4] Bano D. Prehn J. H. M. (2018). Apoptosis-inducing factor (AIF) in physiology and disease: the tale of a repented natural born killer. *EBioMedicine* 30 29–37. 10.1016/j.ebiom.2018.03.016 29605508 PMC5952348

[B5] Bates M. G. D. Newman J. H. Jakovljevic D. G. Hollingsworth K. G. Alston C. L. Zalewski P. (2013). Defining cardiac adaptations and safety of endurance training in patients with m.3243A > G-related mitochondrial disease. *Int. J. Cardiol.* 168 3599–3608. 10.1016/j.ijcard.2013.05.062 23742928 PMC3819621

[B6] Bénit P. Goncalves S. Dassa E. P. Brière J. J. Rustin P. (2008). The variability of the Harlequin mouse phenotype resembles that of human mitochondrial-complex I-deficiency syndromes. *PLoS One* 3:e0003208. 10.1371/journal.pone.0003208 18791645 PMC2527683

[B7] Bird M. J. Thorburn D. R. Frazier A. E. (2014). Modelling biochemical features of mitochondrial neuropathology. *Biochim. Biophys. Acta Gen. Subj.* 1840 1380–1392. 10.1016/j.bbagen.2013.10.017 24161927

[B8] Cabral D. F. Rice J. Morris T. P. Rundek T. Pascual-Leone A. Gomes-Osman J. (2019). Exercise for brain health: an investigation into the underlying mechanisms guided by dose. *Neurotherapeutics* 16 580–599. 10.1007/s13311-019-00749-w 31197642 PMC6694330

[B9] Camiletti-Moirón D. Aparicio V. A. Aranda P. Radak Z. (2013). Does exercise reduce brain oxidative stress? A systematic review. *Scand. J. Med. Sci. Sport.* 23 202–212. 10.1111/sms.12065 23495801

[B10] Cejudo P. Bautista J. Montemayor T. Villagómez R. Jiménez L. Ortega F. (2005). Exercise training in mitochondrial myopathy: a randomized controlled trial. *Muscle Nerve* 32 342–350. 10.1002/mus.20368 15962332

[B11] Chinopoulos C. Adam-Vizi V. (2010). Mitochondria as ATP consumers in cellular pathology. *Biochim. Biophys. Acta Mol. Basis Dis.* 1802 221–227. 10.1016/j.bbadis.2009.08.008 19715757

[B12] Clark-Matott J. Saleem A. Dai Y. Shurubor Y. Ma X. Safdar A. (2015). Metabolomic analysis of exercise effects in the POLG mitochondrial DNA mutator mouse brain. *Neurobiol. Aging* 36 2972–2983. 10.1016/j.neurobiolaging.2015.07.020 26294258 PMC4609600

[B13] Delezie J. Handschin C. (2018). Endocrine crosstalk between Skeletal muscle and the brain. *Front. Neurol.* 9:698. 10.3389/fneur.2018.00698 30197620 PMC6117390

[B14] Do K. Laing B. T. Landry T. Bunner W. Mersaud N. Matsubara T. (2018). The effects of exercise on hypothalamic neurodegeneration of Alzheimer’s disease mouse model. *PLoS One* 13:e0190205. 10.1371/journal.pone.0190205 29293568 PMC5749759

[B15] Dobson P. F. Rocha M. C. Grady J. P. Chrysostomou A. Hipps D. Watson S. (2016). Unique quadruple immunofluorescence assay demonstrates mitochondrial respiratory chain dysfunction in osteoblasts of aged and Polg A mice. *Sci. Rep.* 6 1–10. 10.1038/srep31907 27553587 PMC4995399

[B16] El Hayek L. Khalifeh M. Zibara V. Assaad R. A. Emmanuel N. El-ghandour R. (2019). Lactate mediates the effects of exercise on learning and memory through SIRT1-dependent activation of hippocampal brain-derived neurotrophic factor (BDNF). *J. Neurosci.* 29 2369–2382. 10.1523/JNEUROSCI.1661-18.2019 30692222 PMC6435829

[B17] Fiuza-Luces C. Díez-Bermejo J. Fernández-De La Torre M. Rodríguez-Romo G. Sanz-Ayán P. Delmiro A. (2018a). Health benefits of an innovative exercise program for mitochondrial disorders. *Med. Sci. Sports Exerc.* 50 1142–1151. 10.1249/MSS.0000000000001546 29315169

[B18] Fiuza-Luces C. Santos-Lozano A. Joyner M. Carrera-Bastos P. Picazo O. Zugaza J. L. (2018b). Exercise benefits in cardiovascular disease: beyond attenuation of traditional risk factors. *Nat. Rev. Cardiol.* 15 731–743. 10.1038/s41569-018-0065-1 30115967

[B19] Fiuza-Luces C. Soares-Miranda L. González-Murillo Á Palacio J. M. Colmenero I. Casco F. (2013). Exercise benefits in chronic graft versus host disease: a murine model study. *Med. Sci. Sports Exerc.* 45 1703–1711. 10.1249/MSS.0b013e31828fa004 23954992

[B20] Fiuza-Luces C. Valenzuela P. L. Laine-Menéndez S. Fernández-de la Torre M. Bermejo-Gómez V. Rufián-Vázquez L. (2019). Physical exercise and mitochondrial disease: insights from a mouse model. *Front. Neurol.* 10:790. 10.3389/fneur.2019.00790 31402893 PMC6673140

[B21] He X. Liu D. Zhang Q. Liang F. Dai G. Zeng J. (2017). Voluntary exercise promotes glymphatic clearance of amyloid beta and reduces the activation of astrocytes and microglia in aged mice. *Front. Mol. Neurosci.* 10:144. 10.3389/fnmol.2017.00144 28579942 PMC5437122

[B22] Hu E. Du H. Zhu X. Wang L. Shang S. Wu X. (2018). Beta-hydroxybutyrate promotes the expression of BDNF in hippocampal neurons under adequate glucose supply. *Neuroscience* 386 315–325. 10.1016/j.neuroscience.2018.06.036 29966721

[B23] Jeppesen T. D. Dunø M. Schwartz M. Krag T. Rafiq J. Wibrand F. (2009). Short- and long-term effects of endurance training in patients with mitochondrial myopathy. *Eur. J. Neurol.* 16 1336–1339. 10.1111/j.1468-1331.2009.02660.x 19486129

[B24] Jeppesen T. D. Schwartz M. Olsen D. B. Wibrand F. Krag T. Dunø M. (2006). Aerobic training is safe and improves exercise capacity in patients with mitochondrial myopathy. *Brain* 129 3402–3412. 10.1093/brain/awl149 16815877

[B25] Jiang T. Zhang L. Pan X. Zheng H. Chen X. Li L. (2017). Physical exercise improves cognitive function together with microglia phenotype modulation and remyelination in chronic cerebral hypoperfusion. *Front. Cell. Neurosci.* 11:404. 10.3389/fncel.2017.00404 29311834 PMC5743796

[B26] Kirby D. M. Thorburn D. R. Turnbull D. M. Taylor R. W. (2007). Biochemical assays of respiratory chain complex activity. *Methods Cell Biol.* 80 93–119. 10.1016/S0091-679X(06)80004-X 17445690

[B27] Klein J. A. Longo-Guess C. M. Rossmann M. P. Seburn K. L. Hurd R. E. Frankel W. N. (2002). The harlequin mouse mutation downregulates apoptosis-inducing factor. *Nature* 419 367–374. 10.1038/nature01034 12353028

[B28] Kohman R. A. Bhattacharya T. K. Wojcik E. Rhodes J. S. (2013). Exercise reduces activation of microglia isolated from hippocampus and brain of aged mice. *J. Neuroinflammation* 10:885. 10.1186/1742-2094-10-114 24044641 PMC3848770

[B29] Lai J. H. Chen K. Y. Wu J. C. C. Olson L. Brené S. Huang C. Z. (2019). Voluntary exercise delays progressive deterioration of markers of metabolism and behavior in a mouse model of Parkinson’s disease. *Brain Res.* 1720 6–13. 10.1016/j.brainres.2019.146301 31226324 PMC6702069

[B30] Lechauve C. Augustin S. Cwerman-Thibault H. Reboussin É Roussel D. Lai-Kuen R. (2014). Neuroglobin gene therapy prevents optic atrophy and preserves durably visual function in harlequin mice. *Mol. Ther.* 22 1096–1109. 10.1038/mt.2014.44 24622090 PMC4048897

[B31] Liu-Ambrose T. Barha C. K. Best J. R. (2018). Physical activity for brain health in older adults. *Appl. Physiol. Nutr. Metab.* 43 1105–1112. 10.1139/apnm-2018-0260 30306793

[B32] Lu Y. Dong Y. Tucker D. Wang R. Ahmed M. E. Brann D. (2017). Treadmill exercise exerts neuroprotection and regulates microglial polarization and oxidative stress in a streptozotocin-induced rat model of sporadic Alzheimer’s disease. *J. Alzheimer’s Dis.* 56 1469–1484. 10.3233/JAD-160869 28157094 PMC5450951

[B33] Medja F. Allouche S. Frachon P. Jardel C. Malgat M. Mousson de Camaret B. (2009). Development and implementation of standardized respiratory chain spectrophotometric assays for clinical diagnosis. *Mitochondrion* 9 331–339. 10.1016/j.mito.2009.05.001 19439198

[B34] Mekala N. K. Kurdys J. Depuydt M. M. Vazquez E. J. Rosca M. G. (2019). Apoptosis inducing factor deficiency causes retinal photoreceptor degeneration. The protective role of the redox compound methylene blue. *Redox Biol.* 20 107–117. 10.1016/j.redox.2018.09.023 30300862 PMC6175772

[B35] Moon H. Y. Becke A. Berron D. Becker B. Sah N. Benoni G. (2016). Running-induced systemic cathepsin B secretion is associated with memory function. *Cell Metab.* 24 332–340. 10.1016/j.cmet.2016.05.025 27345423 PMC6029441

[B36] Murphy J. L. Blakely E. L. Schaefer A. M. He L. Wyrick P. Haller R. G. (2008). Resistance training in patients with single, large-scale deletions of mitochondrial DNA. *Brain* 131 2832–2840. 10.1093/brain/awn252 18984605

[B37] Ng Y. S. Turnbull D. M. (2016). Mitochondrial disease: genetics and management. *J. Neurol.* 263 179–191. 10.1007/s00415-015-7884-3 26315846 PMC4723631

[B38] Oliveira L. A. S. D. Martins C. P. Horsczaruk C. H. R. Silva D. C. L. D. Vasconcellos L. F. Lopes A. J. (2018). Partial body weight-supported treadmill training in spinocerebellar ataxia. *Rehabil. Res. Pract.* 2018 1–8. 10.1155/2018/7172686 29535874 PMC5817333

[B39] Pinho R. A. Aguiar A. S. Radák Z. (2019). Effects of resistance exercise on cerebral redox regulation and cognition: an interplay between muscle and brain. *Antioxidants* 8:529 10.3390/antiox811052 PMC6912783 31698763

[B40] Ross J. M. Coppotelli G. Branca R. M. Kim K. M. Lehtiö J. Sinclair D. A. (2019). Voluntary exercise normalizes the proteomic landscape in muscle and brain and improves the phenotype of progeroid mice. *Aging Cell* 18:e13029. 10.1111/acel.13029 31489782 PMC6826127

[B41] Safdar A. Bourgeois J. M. Ogborn D. I. Little J. P. Hettinga B. P. Akhtar M. (2011). Endurance exercise rescues progeroid aging and induces systemic mitochondrial rejuvenation in mtDNA mutator mice. *Proc. Natl. Acad. Sci. U.S.A.* 108 4135–4140. 10.1073/pnas.1019581108 21368114 PMC3053975

[B42] Safdar A. Khrapko K. Flynn J. M. Saleem A. De Lisio M. Johnston A. P. W. (2016). Exercise-induced mitochondrial p53 repairs mtDNA mutations in mutator mice. *Skelet. Muscle* 6:7. 10.1186/s13395-016-0075-9 26834962 PMC4733510

[B43] Schiff M. Bénit P. El-Khoury R. Schlemmer D. Benoist J. F. Rustin P. (2011). Mouse studies to shape clinical trials for mitochondrial diseases: high fat diet in Harlequin mice. *PLoS One* 6:e0028823. 10.1371/journal.pone.0028823 22174907 PMC3236768

[B44] Siciliano G. Manca M. L. Renna M. Prontera C. Mercuri A. Murri L. (2000). Effects of aerobic training on lactate and catecholaminergic exercise responses in mitochondrial myopathies. *Neuromuscul. Disord.* 10 40–45. 10.1016/s0960-8966(99)00068-1 10677862

[B45] Siciliano G. Simoncini C. Gerfo A. L. Orsucci D. Ricci G. Mancuso M. (2012). Effects of aerobic training on exercise-related oxidative stress in mitochondrial myopathies. *Neuromuscul. Disord.* 22 S172–S177. 10.1016/j.nmd.2012.10.005 23182634 PMC3526792

[B46] Sleiman S. F. Henry J. Al-Haddad R. El Hayek L. Haidar E. A. Stringer T. (2016). Exercise promotes the expression of brain derived neurotrophic factor (BDNF) through the action of the ketone body β- hydroxybutyrate. *eLife* 5:e15092. 10.7554/eLife.15092 27253067 PMC4915811

[B47] Stambolsky P. Weisz L. Shats I. Klein Y. Goldfinger N. Oren M. (2006). Regulation of AIF expression by p53. *Cell Death Differ.* 13 2140–2149. 10.1038/sj.cdd.4401965 16729031

[B48] Taivassalo T. De Stefano N. Argov Z. Matthews P. M. Chen J. Genge A. (1998). Effects of aerobic training in patients with mitochondrial myopathies. *Neurology* 50 1055–1060. 10.1212/WNL.50.4.1055 9566394

[B49] Taivassalo T. De Stefano N. Chen J. Karpati G. Arnold D. L. Argov Z. (1999). Short-term aerobic training response in chronic myopathies. *Muscle Nerve* 22 1239–1243. 10.1002/(sici)1097-4598(199909)22:9<1239::aid-mus11>3.0.co;2-w 10454720

[B50] Taivassalo T. Gardner J. L. Taylor R. W. Schaefer A. M. Newman J. Barron M. J. (2006). Endurance training and detraining in mitochondrial myopathies due to single large-scale mtDNA deletions. *Brain* 129 3391–3401. 10.1093/brain/awl282 17085458

[B51] Taivassalo T. Matthews P. M. De Stefano N. Sripathi N. Genge A. Karpati G. (1996). Combined aerobic training and dichloroacetate improve exercise capacity and indices of aerobic metabolism in muscle cytochrome oxidase deficiency. *Neurology* 47 529–534. 10.1212/WNL.47.2.529 8757032

[B52] Taivassalo T. Shoubridge E. A. Chen J. Kennaway N. G. DiMauro S. Arnold D. L. (2001). Aerobic conditioning in patients with mitochondrial myopathies: physiological, biochemical, and genetic effects. *Ann. Neurol.* 50 133–141. 10.1002/ana.1050 11506394

[B53] Trifunovic A. Wredenberg A. Falkenberg M. Spelbrink J. N. Rovio A. T. Bruder C. E. (2004). Premature ageing in mice expressing defective mitochondrial DNA polymerase. *Nature* 429 417–423. 10.1038/nature02517 15164064

[B54] Trounce I. A. Kim Y. L. Jun A. S. Wallace D. C. (1996). [42] Assessment of mitochondrial oxidative phosphorylation in patient muscle biopsies, lymphoblasts, and transmitochondrial cell lines. *Methods Enzymol.* 264 484–509. 10.1016/s0076-6879(96)64044-0 8965721

[B55] Tucker E. J. Compton A. G. Calvo S. E. Thorburn D. R. (2011). The molecular basis of human complex i deficiency. *IUBMB Life* 63 669–677. 10.1002/iub.495 21766414

[B56] Vahsen N. Candé C. Brière J. J. Bénit P. Joza N. Larochette N. (2004). AIF deficiency compromises oxidative phosphorylation. *EMBO J.* 23 4679–4689. 10.1038/sj.emboj.7600461 15526035 PMC533047

[B57] Valenzuela P. L. Castillo-García A. Morales J. S. de la Villa P. Hampel H. Emanuele E. (2020). Exercise benefits on Alzheimer’s disease: state-of-the-science. *Ageing Res. Rev.* 62:101108. 10.1016/j.arr.2020.101108 32561386

[B58] Wang R. Y. Huang F. Y. Soong B. W. Huang S. F. Yang Y. R. (2018). A randomized controlled pilot trial of game-based training in individuals with spinocerebellar ataxia type 3. *Sci. Rep.* 8 1–7. 10.1038/s41598-018-26109-w 29777115 PMC5959926

[B59] World Health Organization. (2010). *WHO Global Recommendations on Physical Activity for Health.* Geneva: WHO. 26180873

[B60] Wrann C. D. White J. P. Salogiannnis J. Laznik-Bogoslavski D. Wu J. Ma D. (2013). Exercise induces hippocampal BDNF through a PGC-1α/FNDC5 pathway. *Cell Metab.* 18 649–659. 10.1016/j.cmet.2013.09.008 24120943 PMC3980968

