# Supplementary material for: Exercise Training and Neurodegeneration in Mitochondrial Disorders: Insights From the Harlequin Mouse
Source: Front Physiol. 2020 Dec 8;11:594223. doi: 10.3389/fphys.2020.594223 (PMC7752860; doi:10.3389/fphys.2020.594223)
Supplement: Supplementary file 1 [file Table_1.DOCX]

**Supplementary Table 1.** Primary antibodies.

| Antibody | Reference | Dilution (WB) | Dilution (IMF) |
| --- | --- | --- | --- |
| Arginase 1 | Abcam ab60176 | 1:5000 |  |
| Calbindin | Abcam ab11426 | 1:10000 | 1:500 |
| Caspase 3 | Cell Signalling #9662 | 1:1000 |  |
| Catalase | Sigma C0979 | 1:1000 |  |
| Citrate synthase | Abcam ab96600 | 1:1000 |  |
| COXI | Abcam ab 14705 |  | 1:200 |
| GAD-65/67 | Sigma G5163 |  | 1:100 |
| GFAP | Abcam ab53554 | 1:1000 |  |
| GFAP | Sigma G3893 |  | 1:500 |
| Glutathione reductase | Abcam ab16801 | 1:1000 |  |
| Grim19 | Abcam ab110240 |  | 1:100 |
| HSP60 | Abcam ab59457 | 1:20000 |  |
| Hydroxynonenal-modified proteins | Abcam ab48506 | 1:50 |  |
| IBA1 | Abcam ab178846 | 1:100 | 1:500 |
| IL-1β | Cell Signalling #12242 | 1:500 |  |
| NDUFA9 | Abcam ab14713 | 1:1000 |  |
| NDUFS1 | GeneTex GTX113787 | 1:1000 |  |
| NeuN | Abcam ab177487 | 1:1000 | 1:500 |
| NRF1 | Abcam ab175932 | 1:2000 |  |
| OXPHOS cocktail | Abcam ab110413 | 1:250 |  |
| p53 | Cell Signalling #2527 | 1:1000 |  |
| Cleaved PARP | Abcam ab32064 | 1:1000 |  |
| Peroxiredoxin 6 | GeneTex GTX115262 | 1:1000 |  |
| Cytosolic SOD | Enzo ADI-SOD-100 | 1:1000 |  |
| Mitochondrial SOD | Millipore 06-984 | 1:1000 |  |
| SDHA | Abcam ab14715 |  | 1:100 |
| TNF-α | Abcam ab1793 | 1:1000 |  |
| γ-tubulin | Sigma T6557 | 1:10000 |  |
| VDAC1 | Abcam ab14734 | 1:1000 |  |
| Vimentin | Abcam ab92547 | 1:1000 |  |

Abbreviations: WB, western blot; IMH, immunohistochemistry.
